# Preoperative Cognitive Function and Physical Frailty Predict Decision Satisfaction and Postoperative Adherence in Older Gynecologic Oncology Patients: A Prospective Observational Study

**DOI:** 10.3390/curroncol33020118

**Published:** 2026-02-17

**Authors:** Celal Akdemir, Merve Konal, Mücahit Furkan Balcı, Gülin Özuyar Şimşek, Zeliha Öcal, Fatih Yıldırım, Zeynep Gül Dağlar, Serkan Karaoğlu, Muzaffer Sancı

**Affiliations:** 1 Department of Gynecologic Oncology, İzmir City Hospital, İzmir 35540, Türkiye; mervekonal@gmail.com (M.K.); ozuyar92@gmail.com (G.Ö.Ş.); drserkank@gmail.com (S.K.); drsanci@yahoo.com (M.S.); 2 Department of Gynecology and Obstetrics, İzmir Torbalı State Hospital, İzmir 35860, Türkiye; mucahitfurkanbalci@gmail.com; 3 Department of Gynecology and Obstetrics, İzmir Tepecik Training and Research Hospital, İzmir 35100, Türkiye; zelihaocal96@hotmail.com (Z.Ö.); fatihyldrm6@gmail.com (F.Y.); 4 Department of Psychiatry, Dokuz Eylül University Faculty of Medicine, İzmir 35390, Türkiye; zeynepguldaglar@gmail.com

**Keywords:** gynecologic oncology, elderly, cognitive assessment, frailty, postoperative recovery, MoCA, CFS

## Abstract

With increasing life expectancy, a growing proportion of patients undergoing surgery for gynecologic cancers are older adults, underscoring the need for reliable predictors of postoperative recovery and patient engagement. Cognitive function and physical frailty are recognized determinants of surgical outcomes, yet their relative impact on patient centered outcomes remains insufficiently explored. This prospective observational study included 68 women aged 65 years and older who underwent abdominal surgery for gynecologic malignancies. Preoperative cognitive function was assessed using the Montreal Cognitive Assessment, and physical frailty was evaluated with the Clinical Frailty Scale. Postoperative outcomes included early recovery parameters, complications, surgical decision satisfaction, and home-based adherence. Higher cognitive scores were associated with earlier mobilization, shorter hospital stay, better postoperative adherence, and greater decision satisfaction, whereas higher frailty scores were associated with delayed recovery and increased complication risk. In regression analyses, preoperative cognitive function was significantly associated with both postoperative adherence and surgical decision satisfaction, whereas physical frailty was not. These findings indicate that preoperative cognitive screening may have predictive value for patient centered recovery behaviors and decision satisfaction in this setting; however, the prediction estimates should be considered exploratory and warrant validation in larger, multicenter cohorts.

## Simple Summary

As the population ages, an increasing number of older women undergo surgery for gynecologic cancers, yet predicting recovery and engagement in postoperative care remains challenging. This study explored whether simple preoperative assessments of cognitive function and physical frailty could help predict how well patients recover after surgery, adhere to recommended care, and feel satisfied with their surgical decision. We found that cognitive function before surgery was a stronger predictor of postoperative recovery behaviors and decision satisfaction than physical frailty. Patients with lower cognitive scores were more likely to experience complications, delayed recovery, and poorer adherence to postoperative recommendations. These findings suggest that routine cognitive screening before surgery may help clinicians identify patients who need additional support, enabling more personalized care and potentially improving postoperative support and outcomes for older women with gynecologic cancers.

## 1. Introduction

Cancer remains one of the leading causes of global mortality, along with cardiovascular diseases. According to GLOBOCAN 2020 data, approximately 10 million people die each year worldwide due to cancer-related causes [1]. By 2040, the incidence of cancer is projected to increase by nearly 47%, largely attributed to the growth of the aging population [2]. Projections from the World Health Organization and the United Nations indicate that, by 2050, the population aged over 65 years will reach 1.6 billion, placing a substantial burden on healthcare systems [3].

Gynecologic cancers are malignancies with increasing incidence, particularly in the postmenopausal period. The mean age at diagnosis for endometrial cancer is 67 years, with the highest incidence observed between 65 and 75 years [4,5]. For ovarian cancer, the median age at diagnosis is 63 years, and approximately half of the cases occur in women aged 60 years or older [6]. Although cervical cancer peaks at younger ages, nearly 20% of cases are diagnosed after the age of 65, and mortality rates are significantly higher in this age group [7,8].

This trend complicates the management of patients diagnosed at an advanced age and highlights the limitations of approaches that rely solely on tumor-specific oncologic parameters in surgical decision making. In the geriatric population, age-related physiological changes, comorbidities, and limited functional reserves are critical determinants that directly influence perioperative outcomes. In particular, cognitive impairment and the degree of physical frailty have been increasingly emphasized in the contemporary literature as significant factors affecting multidimensional outcomes such as postoperative complication risk, length of hospital stay, recovery after discharge, treatment adherence, and overall patient satisfaction.

In geriatric oncology patients, the impact of cognitive and physical status on surgical outcomes is gaining increasing importance. The Montreal Cognitive Assessment (MoCA) provides high sensitivity for the early detection of mild cognitive impairment, enabling an objective evaluation of decision-making capacity [9]. The Clinical Frailty Scale (CFS), on the other hand, offers a rapid, practical, and prognostically valuable assessment of physical reserve and frailty level [10]. For instance, Chatmongkolchart et al. reported that CFS independently predicted postoperative complications in elderly patients undergoing non-cardiac surgery, and its predictive accuracy was enhanced when combined with nutritional status [11]. Similarly, a systematic review by Di Donato et al. in gynecologic oncology patients highlighted preoperative frailty assessment as a critical determinant for predicting adverse surgical outcomes and survival [12].

The combined assessment of MoCA and CFS may provide predictive value not only for postoperative complication risk but also for pre-discharge recovery indicators such as mobilization, Triflow use, and oral intake, as well as home-based postoperative adherence parameters encompassing medication compliance, exercise participation, and independent living. This prospective study aimed to evaluate the relationship between preoperative cognitive function (MoCA) and physical frailty (CFS) with surgical decision satisfaction and postoperative adherence in geriatric gynecologic oncology patients. We hypothesized that patients with lower MoCA or higher CFS scores would demonstrate lower surgical decision satisfaction and poorer postoperative adherence. The findings of this study may provide preliminary evidence supporting the integration of cognitive and frailty assessments into the preoperative evaluation of geriatric gynecologic oncology patients.

The primary objective of this prospective observational study was to evaluate the association between preoperative cognitive function (MoCA) and physical frailty (CFS) with surgical decision satisfaction and postoperative adherence in geriatric gynecologic oncology patients.

## 2. Materials and Methods

### 2.1. Study Design and Ethical Approval

This study was designed as a prospective observational study to test the hypothesis that preoperative cognitive function (MoCA) and physical frailty (CFS) are associated with surgical decision satisfaction and postoperative adherence in geriatric gynecologic oncology patients. The study protocol was conducted in accordance with the principles of the Declaration of Helsinki and approved by the Non-Interventional Ethics Committee of İzmir City Hospital (Approval No: 2025/221, Date: 7 May 2025). Written informed consent was obtained from all participants. The study was registered on the ClinicalTrials.gov platform (Identifier: NCT07085000).

### 2.2. Study Population and Surgical Procedures

Between May 2025 and August 2025, women aged ≥65 years with a diagnosis of gynecologic cancer who underwent surgery at the Department of Gynecologic Oncology, İzmir City Hospital, were enrolled in the study. Inclusion criteria were: indication for planned gynecologic oncology surgery, ability to communicate adequately for cognitive evaluation, and sufficient cognitive capacity to provide written informed consent.

Exclusion criteria were: emergency surgical indication, preoperative delirium or major psychiatric disorder defined as a clinically diagnosed condition according to DSM-5 criteria (e.g., major depressive disorder or psychotic disorders), severe sensory impairment precluding MoCA or CFS assessment, and inability to provide informed consent due to cognitive insufficiency. To minimize variability related to surgical technique, only patients undergoing abdominal surgery (laparotomy) were included. All aprocedures were performed by gynecologic oncologists. The standard surgical approach consisted of total abdominal hysterectomy and bilateral salpingo-oophorectomy. Based on intraoperative frozen section results, pelvic and/or para-aortic lymphadenectomy was performed when indicated, omentectomy was added in appropriate cases, and, in patients with cervical cancer, radical hysterectomy with pelvic lymphadenectomy was undertaken.

Eligible patients were consecutively recruited during the study period. All inclusion and exclusion criteria were applied prospectively, and reasons for non-inclusion were recorded at the time of enrollment.

### 2.3. Preoperative Assessment

Demographic information (age, diagnosis, educational level), comorbidity status, and preoperative cognitive and physical parameters (adjusted MoCA score and CFS score) were systematically collected for all patients enrolled in the study.

All patients underwent a preoperative cognitive and frailty evaluation 24–48 h prior to surgery. Cognitive function was assessed using the Montreal Cognitive Assessment (MoCA), a multidimensional, standardized screening tool evaluating attention, memory, executive functions, language, and visuoconstructive abilities, and it was administered by a clinician trained in geriatric assessment. During administration, patients’ concentration, comprehension, and response adequacy were closely monitored to ensure validity of the process. In accordance with the original guidelines, one additional point was assigned for individuals with ≤12 years of formal education [9]. A Turkish validation study confirmed its appropriate psychometric properties and discriminative performance in Turkish populations [13]. MoCA scores were treated as continuous variables and analyzed in correlation and regression models with postoperative surgical decision satisfaction and adherence scores.

Physical frailty was assessed using the Clinical Frailty Scale (CFS), which ranges from 1 (very fit) to 9 (terminally ill). In this study, patients were categorized into three groups based on their CFS scores: “fit (non-frail)” (scores 1–3), “pre-frail” (score 4), and “frail” (scores ≥ 5). Assessments were performed by a physician trained in geriatric evaluation. Administration of the Montreal Cognitive Assessment required approximately 10 min per patient, whereas the Clinical Frailty Scale assessment required less than 2 min, allowing rapid integration into routine preoperative evaluation.

### 2.4. Postoperative Clinical Data

In the early postoperative period, the following parameters were recorded: time to first mobilization (hours), time to first oral intake, length of hospital stay (days), severity of postoperative nausea and vomiting (PONV), and occurrence of complications. During postoperative follow-up, PONV, respiratory exercise (Triflow) adherence, and mobilization behaviors were systematically assessed.

Postoperative nausea and vomiting (PONV) was categorized into four levels based on clinical severity: no symptoms, mild symptoms controlled with a single antiemetic dose, moderate symptoms requiring multiple antiemetics or presenting with both nausea and vomiting, and severe symptoms that remained uncontrolled despite intravenous antiemetic therapy. Triflow adherence was evaluated according to the regularity of practice and classified as non-adherent (never performed), partially adherent (performed for 1–2 days only), or regularly adherent (performed at least three sets daily throughout hospitalization). Mobilization behavior was classified based on the proportion of daily targets achieved, with high adherence defined as mobilization within the first 24 h postoperatively, walking at least three times per day, and achieving more than 80% of daily goals; moderate adherence as achieving 50–80% of daily goals; and low adherence as achieving less than 50% of daily goals. These parameters were monitored and recorded according to standardized protocols by trained members of the research team who were not involved in the preoperative MoCA and CFS assessments.

Postoperative complications were initially evaluated in a binary manner (present or absent) and subsequently categorized using the Clavien–Dindo classification as minor (Grade I–II), managed conservatively, or major (Grade ≥ III), requiring surgical, invasive, or intensive care interventions. Functional status at discharge was assessed based on independence in basic self-care activities, including ambulation, toileting, and dressing, and patients were classified as independent, assisted, or dependent accordingly.

Early postoperative parameters assessed during hospitalization, including Triflow use and mobilization behavior, were recorded as objective clinical recovery indicators. These measures were subsequently used as reference points for the postoperative adherence score, which was calculated at the postoperative day-15 follow-up visit using a Likert-based composite approach reflecting sustained recovery behaviors after discharge.

### 2.5. Postoperative Adherence and Surgical Decision Satisfaction

At the postoperative day-15 outpatient follow-up visit, patients were evaluated for both postoperative adherence and satisfaction with the surgical decision. For patients who had not been discharged by postoperative day 15, the assessments were conducted in-person during inpatient follow-up using the same standardized procedures to ensure consistency of evaluation.

Surgical decision satisfaction was measured using a five-item Likert-type instrument adapted from the Decision Regret Scale (DRS), incorporating questions related to perceived appropriateness of the decision, regret or doubt regarding the outcome, and hypothetical preferences for alternative choices. After reverse-scoring negatively phrased items, a total score was calculated and normalized to a 0–100 scale, with higher scores indicating greater satisfaction and lower decision regret. The original DRS was developed and validated by Brehaut et al. [14], and wording modifications were limited to contextual adaptation for surgical oncology without altering the psychometric structure.

Postoperative adherence was assessed as a multidimensional behavioral construct encompassing five domains relevant to perioperative recovery: frequency of Triflow respiratory exercises, ambulation patterns, medication adherence, independence in self-care activities, and pace of return to daily living activities. These data were derived from a combination of direct in-hospital observation, outpatient follow-up, and caregiver or companion corroboration to reduce sole reliance on patient self-report. Each domain was scored from 1 (minimal adherence) to 5 (optimal adherence) based on predefined behavioral criteria. The summed raw score (range 5–25) was multiplied by four to generate a normalized 0–100 adherence score. For analytical purposes, adherence was categorized as low (<60), moderate (60–80), or high (>80). The full scoring rubric for each domain and score level is provided in Supplementary Table S1. Internal consistency of the composite postoperative adherence score was assessed using Cronbach’s alpha, which demonstrated acceptable reliability (Cronbach’s alpha = 0.84).

Sample size considerations were informed by preliminary data obtained from the first 40 participants, in whom a strong positive correlation was observed between MoCA score and surgical decision satisfaction (r = 0.68, *p* < 0.001). Using GPower version 3.1.9.7, this effect size indicated that a minimum sample size of approximately 13–15 participants would be sufficient to detect this single correlation with a two-sided alpha of 0.05 and 80% power. However, given the broader analytic aims of the study, including multiple correlations, regression models, and subgroup comparisons (for example, according to complication status), we planned to recruit a substantially larger cohort. The final analysis was conducted with 68 patients, providing robust power for the primary correlation analyses while rendering the more complex and subgroup analyses exploratory in nature.

**Figure 1:**
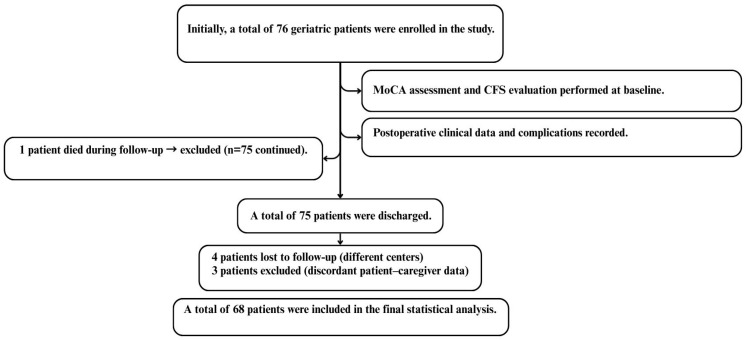
Study flowchart of patient selection, exclusion criteria, and final sample included in the analysis.

Of the 76 participants initially enrolled, four were excluded due to postoperative follow-up occurring at external institutions, which precluded standardized assessment of satisfaction and adherence outcomes. Three additional patients were excluded due to major discrepancies between patient and caregiver reports that conflicted with predefined adjudication criteria, and one patient died in the early postoperative period. As a result, 68 patients were included in the final analysis of postoperative adherence and surgical decision satisfaction (Figure 1).

### 2.6. Statistical Analysis

The data were analyzed using IBM SPSS Statistics for Mac, version 29.0 (IBM Corp., Armonk, NY, USA). Continuous variables were presented as mean ± standard deviation or median (minimum–maximum), and categorical variables were summarized as frequencies (*n*) and percentages (%). Between-group comparisons were performed using the Student’s *t*-test or the Mann–Whitney U test based on the distribution of variables, while categorical variables were compared using the Chi-square test.

Surgical decision satisfaction and postoperative adherence scores were treated as continuous variables to preserve statistical power and avoid information loss. Categorized adherence levels (low, moderate, high) were used only for descriptive and subgroup comparison purposes. Associations between MoCA and CFS scores with decision satisfaction and postoperative adherence were evaluated using Pearson or Spearman correlation tests, as appropriate.

To further explore relationships between variables, linear regression models were constructed. Given the sample size and the strong collinearity between MoCA and CFS scores (r = −0.62), regression analyses were performed using separate models to avoid multicollinearity and overfitting. Model performance was reported using unstandardized β coefficients and the coefficient of determination (R^2^). A two-sided *p* < 0.05 was considered statistically significant.

To minimize observer bias, the assessors evaluating postoperative satisfaction and adherence outcomes were blinded to baseline MoCA and CFS scores. Adherence assessments were performed by trained members of the clinical research team using a predefined standardized protocol, with caregiver corroboration; formal inter-rater agreement statistics were not calculated. Given the exploratory nature of the study and the relatively small sample size, multiple comparisons adjustment was not applied. Instead, emphasis was placed on effect sizes and consistent directionality across related outcomes. In addition, given the limited sample size and low number of complication events, regression and ROC analyses were performed in an exploratory, hypothesis-generating framework and were not intended for definitive prediction modeling. Potential confounding variables such as age, comorbidity burden, and ASA classification were examined in univariate analyses, and variables showing clinical relevance were considered in exploratory modeling.

## 3. Results

### 3.1. Patient Characteristics

In total, 68 older patients with gynecologic malignancies were included in the study. The mean age was 71.5 ± 4.9 years (range: 65–88), and the mean body mass index (BMI) was 27.2 ± 3.9. Regarding cancer type, endometrial cancer was the most common diagnosis (64.7%, *n* = 44), followed by ovarian cancer (32.4%, *n* = 22) and cervical cancer (2.9%, *n* = 2).

Most patients (82.3%) had ≤12 years of education, and the overall comorbidity rate was 94%. The most frequent comorbidities were hypertension (54.4%), diabetes mellitus (30.9%), coronary artery disease or ischemic heart disease (19.1%), chronic pulmonary disease including COPD or asthma (17.6%), chronic kidney disease (7.4%), and a history of cerebrovascular disease (4.4%). A substantial proportion of patients had more than one comorbidity. According to the American Society of Anesthesiologists (ASA) classification, 58.8% (*n* = 40) were classified as ASA I–II and 41.2% (*n* = 28) as ASA ≥ III.

The mean Montreal Cognitive Assessment (MoCA) score was 20.9 ± 3.8, and the mean Clinical Frailty Scale (CFS) score was 3.7 ± 1.4. Based on CFS categories, 45.6% of patients were classified as fit, 29.4% as pre-frail, and 25.0% as frail. The mean surgical decision satisfaction score (normalized to a 0–100 scale) was 74.4 ± 13.9, and the mean postoperative adherence score was 75.9 ± 14.0 (Table 1).

### 3.2. Early Postoperative Outcomes

The mean time to first mobilization was 12.2 ± 6.3 h, and the mean time to first oral intake was 13.8 ± 6.3 h. Regular Triflow exercise adherence during hospitalization was observed in 27.9% of patients. The mean length of hospital stay was 5.5 ± 4.5 days.

Postoperative nausea and vomiting (PONV) occurred in 27.9% of patients, including mild symptoms in 19.1%, moderate symptoms in 7.3%, and severe symptoms in 1.5%. Mobilization adherence was classified as high in 50.0% of patients, moderate in 32.3%, and low in 17.7%. At discharge, 51.5% of patients were independent in basic self-care activities, 36.8% required assistance, and 11.8% were dependent.

### 3.3. Correlations with Clinical Outcomes

Higher MoCA scores were significantly associated with earlier mobilization (r = −0.59, *p* < 0.001), earlier oral intake (r = −0.41, *p* < 0.001), better Triflow adherence (ρ = 0.56, *p* < 0.001), shorter hospital stay (r = −0.58, *p* < 0.001), and greater functional independence at discharge (ρ = −0.69, *p* < 0.001).

Conversely, higher CFS scores were correlated with delayed mobilization (ρ = 0.43, *p* < 0.001), delayed oral intake (ρ = 0.27, *p* = 0.023), longer hospital stay (ρ = 0.34, *p* = 0.004), and greater dependency at discharge (ρ = 0.53, *p* < 0.001). The association between CFS score and Triflow adherence was weak and borderline significant (ρ = −0.24, *p* = 0.053). No significant association was observed between CFS score and additional analgesic use (*p* = 0.491) (Table 2).

### 3.4. Postoperative Complications

The overall postoperative complication rate was 22.1% (15/68). Minor complications (Clavien–Dindo Grade I–II) occurred in 19.1% of patients, while major complications (Grade ≥ III) were observed in 2.9%.

Patients who developed complications had significantly lower MoCA scores (17.3 ± 4.1 vs. 21.9 ± 3.0; *p* < 0.001) and higher CFS scores (4.7 ± 1.7 vs. 3.4 ± 1.1; *p* < 0.05). Length of hospital stay was significantly longer in the complication group (9.5 ± 7.6 vs. 4.4 ± 2.2 days; *p* < 0.01), and surgical decision satisfaction scores were significantly lower (67.1 ± 13.7 vs. 76.8 ± 13.0; *p* < 0.05). In addition, regular Triflow exercise adherence (6.7% vs. 34.0%, *p* < 0.05) and high mobilization adherence (20.0% vs. 58.5%, *p* < 0.001) were markedly reduced among patients with complications (Table 3).

### 3.5. ROC Curve Analysis

**Figure 2:**
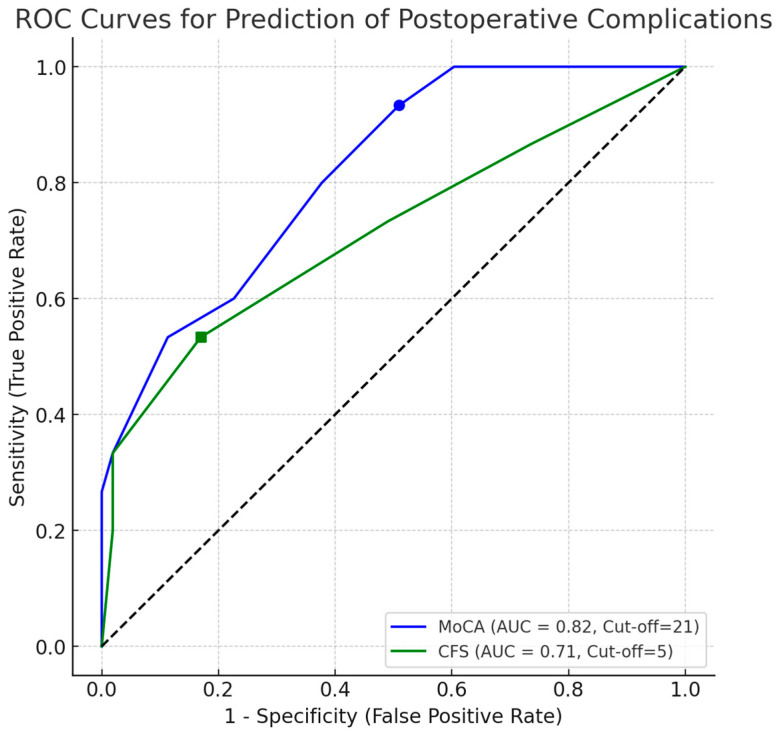
Receiver operating characteristic (ROC) curves for the predictive performance of MoCA and CFS scores in relation to postoperative complications. The dashed line represents the line of no discrimination (AUC = 0.5).

Receiver operating characteristic (ROC) curve analysis demonstrated that MoCA showed strong discrimination for predicting postoperative complications (AUC = 0.82; 95% CI: 0.71–0.92; cut-off value = 21; sensitivity 93.3%, specificity 49.1). In contrast, CFS demonstrated moderate discriminatory performance (AUC = 0.71; 95% CI: 0.57–0.84; cut-off value = 5; sensitivity 53.3%, specificity 83.0) (Figure 2).

The selected MoCA cut off prioritized sensitivity and should be viewed as an exploratory screening threshold; given the limited number of events, these estimates require validation before any clinical application.

### 3.6. Associations with Surgical Decision Satisfaction and Postoperative Adherence

MoCA scores were strongly and positively correlated with surgical decision satisfaction (r = 0.70, *p* < 0.001) and postoperative adherence (r = 0.73, *p* < 0.001). Conversely, CFS scores were moderately and negatively correlated with surgical decision satisfaction (r = −0.45, *p* < 0.001) and postoperative adherence (r = −0.43, *p* < 0.001). A significant inverse correlation was also observed between MoCA and CFS scores (r = −0.62, *p* < 0.001) (Table 4).

### 3.7. Regression Analysis

Linear regression analysis identified MoCA score as a statistically significant predictor within the exploratory model of both postoperative adherence and surgical decision satisfaction. Each one-point increase in MoCA score was associated with an approximately 2.8-point increase in the postoperative adherence score (0–100 scale) (β = 2.78, *p* < 0.001) and a 2.5-point increase in the surgical decision satisfaction score (β = 2.55, *p* < 0.001). CFS score was not a significant predictor in these models (*p* > 0.05).

MoCA and CFS were not entered simultaneously into regression models due to collinearity; therefore, separate models were evaluated (Table 5).

Linear regression analysis: only the MoCA score was included as a predictor. The models explained approximately 49% of the variance in surgical decision satisfaction (R^2^ = 0.49) and 53% of the variance in postoperative adherence (R^2^ = 0.53). β represents unstandardized regression coefficients. A 1-point increase in MoCA score is associated with a 2.78-point increase in the postoperative adherence score (0–100 scale) and a 2.55-point increase in the surgical decision satisfaction score. MoCA and CFS scores were not entered simultaneously into the regression models due to collinearity; therefore, separate models were evaluated.

## 4. Discussion

This prospective observational study evaluated the relationship between preoperative cognitive and physical frailty status and postoperative outcomes in geriatric gynecologic oncology patients undergoing laparotomy. Higher MoCA scores were significantly associated with improved postoperative adherence and greater surgical decision satisfaction both before and after discharge. Conversely, elevated CFS scores correlated with delayed mobilization, increased complication rates, and greater functional dependency. A significant negative correlation was observed between cognitive and frailty scores. Importantly, regression analyses revealed that only the MoCA score was significantly associated with both clinical outcomes in regression models. The predominant influence of MoCA may reflect the central role of cognitive function in directly modulating both complication risk and patient behaviors, including treatment adherence and decision making. In contrast, the impact of CFS appears to occur more indirectly, mediated through physical capacity. Notably, patients who developed postoperative complications had significantly lower MoCA scores and higher CFS scores, suggesting that both reduced cognitive capacity and increased frailty may contribute to adverse outcomes.

Previous studies have emphasized the prognostic significance of cognitive and physical frailty in surgical outcomes among elderly patients. In a systematic review by Di Donato et al., preoperative frailty assessment in elderly patients undergoing gynecologic oncology surgery was identified as a strong predictor of complications, intensive care needs, and discharge to facilities other than home [12]. Similarly, a prospective cohort study by Fang et al. demonstrated that the combined assessment of cognitive and physical frailty, described as cognitive frailty, significantly increased the risk of postoperative complications and functional decline [15]. In the broader surgical literature, physical frailty has been strongly linked to long-term mortality, while diminished cognitive reserve has been noted to adversely impact treatment adherence [16]. Taken together, these findings highlight the clinical relevance of incorporating both cognitive and frailty assessments into preoperative evaluation. In particular, patients with MoCA ≤ 21 and CFS ≥ 5 may represent a subgroup warranting further investigation regarding targeted perioperative support strategies.

Recent data in oncogeriatrics increasingly emphasize the importance of cognitive and behavioral dimensions in perioperative recovery. In gynecologic oncology, multi-institutional ERAS cohorts have shown that protocol adherence and active patient participation significantly influence postoperative outcomes beyond traditional surgical complexity metrics [17]. Moreover, studies in older cancer survivors have demonstrated that cognitive impairment is closely associated with functional decline, frailty progression, and reduced engagement in self-management and health-related behaviors [18]. In the broader perioperative literature, preoperative cognitive impairment has been identified as an independent determinant of postoperative delirium and neurocognitive complications, underscoring its relevance for perioperative risk stratification [19]. Taken together, these findings support the integration of cognitive screening into perioperative pathways and prehabilitation models for older surgical patients.

In this context, the strong correlation observed between MoCA scores and both postoperative adherence and surgical decision satisfaction in our study supports the notion that cognitive capacity may play an important role not only in relation to complication risk but also in facilitating active patient participation in the treatment process and interaction with healthcare services.

In the subgroup analysis according to complications, both cognitive and physical frailty levels were found to be significantly associated with clinical outcomes. This group also demonstrated significantly lower scores for surgical decision satisfaction and postoperative adherence. Consistent with our findings, previous studies have reported that the presence of cognitive impairment and physical frailty is associated with increased complication rates and higher readmission frequencies after discharge [20,21]. Despite the limited sample size and the small number of patients in the major complication subgroup, the statistically significant differences observed between MoCA scores, reflecting cognitive capacity, and CFS scores, reflecting physical frailty, suggest that both components of frailty may play a decisive role in the development of postoperative complications. These results support the concept that the combined evaluation of cognitive and physical parameters may contribute to a more comprehensive exploratory risk assessment framework.

In our study, 50.0% of patients (34/68) demonstrated high adherence to mobilization, while 27.9% (19/68) regularly performed Triflow exercises. In the non-complication group, both mobilization adherence (58.5% vs. 20.0%, *p* < 0.001) and Triflow adherence (34.0% vs. 6.7%, *p* < 0.05) were significantly higher. These findings highlight the critical role of mobilization adherence in preventing complications during the postoperative course.

In our study, the mean MoCA scores were below the commonly accepted cut-off value, suggesting that a proportion of participants may have been within the spectrum of mild cognitive impairment. This raises the possibility of bias in self-reported assessments of postoperative adherence to home-based behaviors. Nevertheless, caregiver input and standardized in-hospital observations partially mitigated this limitation. In particular, deficits in attention, executive function, and memory may lead to non-adherence in fundamental aspects of care such as mobilization, Triflow exercises, and medication use [9,22].

The literature similarly emphasizes that self-reported health questionnaires have limited validity in older adults with cognitive impairment and should be supplemented with observation-based measures [23,24]. Moreover, a meta-analysis by Salari et al. reported a prevalence of mild cognitive impairment of 17.1% in the elderly population, highlighting its significant impact on healthcare utilization and functional independence [25]. These findings indicate that cognitive assessment in geriatric oncology patients may predict not only complication risk but also patient-centered outcomes such as postoperative adherence and surgical decision satisfaction.

Previous studies have also demonstrated that cognitive deficits reduce participation in postoperative rehabilitation, delay functional recovery, and increase the need for home care. Furthermore, in older adults with non-Alzheimer cognitive disorders, cognitive status has been identified as a determinant of engagement in health management and patient education [26,27].

In our study, a significant inverse correlation was identified between cognitive function and physical frailty levels (r = −0.62). Lower MoCA scores were associated with higher CFS scores. This finding is consistent with the literature. For instance, Boyle et al. reported that the development of physical frailty increased the risk of mild cognitive impairment by 63%. Similarly, in a 20-year longitudinal study, Givan et al. demonstrated that individuals with cognitive impairment experienced a significant decline in physical functions [28]. Cognitive and physical frailty are believed to be linked through shared biological mechanisms such as inflammation, sarcopenia, and vascular changes. The frailty phenotype described by Fried et al. conceptually supports the notion that cognitive and physical frailty may progress in parallel but manifest differently in clinical outcomes [29].

In our study, the CFS score was negatively associated with surgical decision satisfaction and postoperative adherence and showed moderate discriminatory power in predicting complications (AUC = 0.71). The proportion of frail individuals was limited to 25% (*n* = 17), with few cases in the advanced frailty group (CFS ≥ 5), which may have reduced the observed effect. Previous studies have shown that advanced physical frailty approximately doubles the risk of postoperative complications [30,31]. Therefore, the limited independent effect of CFS in our study may be attributable both to the distribution of the sample and to the dominant impact of cognitive capacity overshadowing the role of physical frailty. Future studies with larger cohorts are warranted to clarify the independent contributions of each factor, potentially through separate regression models or interaction analyses.

In this study, postoperative adherence was evaluated through multidimensional parameters, including regular performance of Triflow exercises, early mobilization, appropriate use of medications, maintenance of self-care, and return to daily activities. Importantly, the assessment was not limited to self-reporting by patients but also incorporated observations from caregivers. This approach aimed to reduce subjectivity in measurements and provide a more objective evaluation of adherence.

The significant and strong positive correlation observed between the Montreal Cognitive Assessment (MoCA) score and postoperative adherence score indicates that cognitive capacity plays a decisive role in postoperative self-efficacy and active engagement in the treatment process. This finding highlights that cognitive functions are not limited to cognitive outcomes alone but also influence behavioral outcomes such as treatment adherence and recovery.

The impact of cognitive capacity on health behaviors has also been demonstrated in various chronic disease populations. For example, Barclay et al. reported significantly lower treatment adherence rates among individuals with mild cognitive impairment [32]. Similarly, Alosco et al. found that cognitive dysfunction in patients with heart failure negatively affected daily living activities and self-care behaviors [33].

In the present study, the strong correlation between the multidimensional postoperative adherence score and MoCA (r = 0.73) further underscores the critical role of cognitive capacity in recovery engagement. Although this adherence score has not been standardized in the literature, its multidimensional structure and strong correlation with MoCA support its internal consistency and practical interpretability; however, external validation is needed.

The consistent associations of the CFS with complications and functional outcomes demonstrate that physical frailty assessment should also not be overlooked in preoperative risk screening. Therefore, combining cognitive and physical assessments may provide a more comprehensive risk stratification in geriatric gynecologic oncology patients.

In addition, unmeasured residual confounding factors such as socioeconomic status, educational level, health literacy, and family or caregiver support may have influenced postoperative adherence and surgical decision satisfaction. These factors are known to affect engagement in health behaviors and access to postoperative support, particularly in older adults. Although these variables were not systematically assessed in the present study, their potential impact should be considered when interpreting the findings.

This study has several limitations that should be acknowledged. First, the relatively small sample size and the single-center design may limit the generalizability of the findings, particularly for subgroup analyses. Notably, the number of patients who developed postoperative complications was limited (*n* = 15), which may have reduced the statistical power to detect independent effects of physical frailty. Second, postoperative adherence was partly assessed using patient-reported measures, which may be prone to reporting bias, especially among individuals with lower cognitive capacity. However, this limitation was mitigated by incorporating caregiver reports and objective in-hospital observations to enhance assessment reliability. In addition, cultural, educational, and socioeconomic factors, which may influence cognitive test performance, health behaviors, and postoperative adherence, were not formally assessed and may have acted as residual confounders. In addition, detailed disease stage information was not systematically incorporated into the analysis, as the primary focus of the study was on preoperative cognitive and frailty status rather than tumor related oncologic characteristics. This may limit the interpretation of postoperative adherence outcomes in relation to disease severity. Third, although the Clinical Frailty Scale was associated with adverse clinical outcomes in univariate analyses, it did not emerge as an independent predictor in regression models. This finding may reflect the distribution of frailty levels within the cohort as well as the dominant influence of cognitive function, as measured by MoCA, on postoperative behaviors and decision satisfaction. Finally, the postoperative adherence score used in this study represents a composite, non-standardized measure. While its multidimensional structure and internal consistency support its practical interpretability, external validation in larger and multicenter cohorts is warranted. In addition, the sample size calculation was based solely on the primary correlation between MoCA and surgical decision satisfaction and was not specifically powered for all secondary endpoints or subgroup analyses, particularly those involving patients with postoperative complications. These results should therefore be interpreted as exploratory. We acknowledge that multiple correlation and subgroup analyses may increase the risk of Type I error. As this study was exploratory and hypothesis-generating, no multiplicity correction (such as Bonferroni or FDR) was applied. Accordingly, the findings should be considered preliminary and require validation in larger confirmatory cohorts. Future research integrating cognitive assessment into prehabilitation and ERAS models may help determine whether targeted interventions can enhance postoperative adherence and reduce complication profiles in frail older oncology patients.

This study has several noteworthy strengths. It is among the few prospective observational studies to examine the relationship between cognitive and physical frailty levels and postoperative behavioral outcomes in geriatric gynecologic oncology patients. The combined evaluation of surgical decision satisfaction and postoperative adherence provided a holistic perspective on the concept of frailty. Furthermore, the use of validated assessment tools such as MoCA and CFS enhanced the reliability of the data, while the incorporation of caregiver observations in addition to patient self-reports reduced subjectivity and strengthened the objectivity of postoperative adherence evaluation. Importantly, the strong correlation identified in the preliminary analysis of the first 40 patients between MoCA and surgical decision satisfaction (r = 0.68, *p* < 0.001) supports that the final sample of 68 patients provided adequate statistical power for primary correlation analyses.

This study was conducted in a single tertiary center, which may limit the generalizability of the findings. Future studies with larger and multicenter cohorts are needed to more clearly disentangle the independent contributions of MoCA and CFS, ideally through advanced statistical methods such as interaction analyses. It should also be acknowledged that self-reported outcomes in patients with reduced cognitive capacity may be prone to bias due to limitations in perception and memory. Nonetheless, the inclusion of caregiver evaluations, consistency with existing literature, and the observation of lower MoCA scores in patients who developed complications collectively support the validity of the findings. Direct observational or digital monitoring methods may further mitigate these limitations in future research. In addition, the study was not specifically powered for regression modeling or ROC-based complication prediction; therefore, these analyses should be interpreted as exploratory and hypothesis generating.

From a clinical standpoint, the growing emphasis on cognitive screening within oncogeriatric pathways and ERAS-based perioperative care further supports the incorporation of brief cognitive tests such as MoCA into routine preoperative assessment in older cancer patients. Identifying cognitive vulnerability preoperatively may allow individualized optimization strategies, targeted communication, and improved postoperative engagement in rehabilitation and self-care programs. From a guideline perspective, these findings support the consideration of brief cognitive screening as part of routine preoperative assessment in older gynecologic oncology patients. Incorporation of tools such as MoCA into existing oncogeriatric and ERAS-based recommendations may help identify patients at risk for poor postoperative adherence and inform individualized perioperative support strategies. However, confirmation in larger, multicenter cohorts is required before formal guideline adoption.

## 5. Conclusions

Despite its limitations, this study suggests that cognitive assessment may represent a potentially useful factor to consider in perioperative planning and recovery among older cancer patients. The findings indicate that cognitive function appeared more strongly associated than physical frailty in this cohort in relation to postoperative adherence and surgical decision satisfaction. Lower MoCA scores were associated with higher complication risk and reduced engagement in treatment and self-care behaviors.

Accordingly, preoperative cognitive assessment may help inform individualized perioperative management strategies in geriatric gynecologic oncology patients. Brief cognitive screening tools such as MoCA may represent a feasible adjunct to routine preoperative evaluation and could contribute to perioperative risk stratification and patient-centered care. However, these findings should be interpreted cautiously and require confirmation in larger, multicenter cohorts before broader clinical implementation. Integration of cognitive assessment within multidisciplinary perioperative pathways may offer a promising approach for optimizing surgical care in the growing older cancer population.

## Figures and Tables

**Table 1:** Demographic and Clinical Characteristics of the Study Population (*n* = 68).

Variable	Value
Sex (Female), *n* (%)	68 (100)
Age (years), mean ± SD (range)	71.5 ± 4.9 (65–88)
BMI (kg/m^2^), mean ± SD	27.2 ± 3.9
Education ≤ 12 years, n (%)	56 (82.3)
ASA 1–2, *n* (%)	40 (58.8)
ASA ≥ 3, *n* (%)	28 (41.2)
Endometrial cancer, n (%)	44 (64.7)
Ovarian cancer, *n* (%)	22 (32.4)
Cervical cancer, *n* (%)	2 (2.9)
MoCA score, mean ± SD	20.9 ± 3.8
CFS score, mean ± SD	3.7 ± 1.4
Fit, *n* (%)	31 (45.6)
Pre-frail, *n* (%)	20 (29.4)
Frail, *n* (%)	17 (25.0)
Surgical decision satisfaction (0–100 scale), mean ± SD	74.4 ± 13.9
Postoperative adherence score (0–100 scale), mean ± SD	75.9 ± 14.0

**Table 2:** Correlation of MoCA and CFS Scores with Functional and Clinical Parameters.

Variable	MoCA (Correlation Coefficient, *p*)	CFS (Correlation Coefficient, *p*)
Mobilization time (hours)	r = −0.59, *p* < 0.001	ρ = 0.43, *p* < 0.001
Oral intake initiation (hours)	r = −0.41, *p* < 0.001	ρ = 0.27, *p* = 0.023
Triflow adherence	ρ = 0.56, *p* < 0.001	ρ = −0.24, *p* = 0.053
Additional analgesic use	ρ = −0.25, *p* = 0.036	ρ = 0.08, *p* = 0.491
Length of hospital stay (days)	r = −0.58, *p* < 0.001	ρ = 0.34, *p* = 0.004
Functional independence at discharge	ρ = −0.69, *p* < 0.001	ρ = 0.53, *p* < 0.001

Pearson correlation was used for continuous variables, and Spearman correlation for ordinal or binary variables. Functional independence at discharge was coded as 1 = independent, 2 = assisted, and 3 = dependent; therefore, negative correlation coefficients indicate better functional independence with higher MoCA scores.

**Table 3:** Clinical Parameters According to Complication Status.

Variable	No Complications (*n* = 53)	Complications (*n* = 15)	*p*-Value
MoCA score (mean ± SD)	21.94 ± 2.98	17.27 ± 4.06	<0.001
CFS score (mean ± SD)	3.43 ± 1.15	4.67 ± 1.72	<0.05
Surgical decision satisfaction (%)	76.8 ± 13.0	67.1 ± 13.7	<0.05
Length of hospital stay (days)	4.36 ± 2.15	9.47 ± 7.56	<0.01
High Triflow adherence (%)	34.0 (18/53)	6.7 (1/15)	<0.05
High mobilization adherence (%)	58.5 (31/53)	20.0 (3/15)	<0.001

Data are presented as mean ± standard deviation or *n* (%).

**Table 4:** Pearson Correlation Analysis Between Key Variables.

Variable Pair	r	*p*
MoCA–Satisfaction	0.70	<0.001
MoCA–Adherence	0.73	<0.001
CFS–Satisfaction	−0.45	<0.001
CFS–Adherence	−0.43	<0.001
MoCA–CFS	−0.62	<0.001

Correlation coefficients were interpreted as follows: r = 0.10–0.29 (weak), 0.30–0.49 (moderate), 0.50–0.69 (moderately strong), and ≥0.70 (strong).

**Table 5:** Significant Predictors in Regression Analysis.

Dependent Variable	Significant Predictor	β (*p*)
Postoperative Adherence	MoCA score	2.78 (*p* < 0.001)
Surgical Decision Satisfaction	MoCA score	2.55 (*p* < 0.001)

## Data Availability

The data supporting the findings of this study are available from the corresponding author upon reasonable request. Data sharing is subject to ethical and privacy considerations.

## Supplementary Materials

The following supporting information can be downloaded at: https://www.mdpi.com/article/10.3390/curroncol33020118/s1, Table S1: Scoring rubric for the postoperative adherence domain.

## Abbreviations

The following abbreviations are used in this manuscript:

ASA	American Society of Anesthesiologists
AUC	Area under the curve
BMI	Body mass index
CFS	Clinical Frailty Scale
CI	Confidence interval
DRS	Decision Regret Scale
MoCA	Montreal Cognitive Assessment
PONV	Postoperative nausea and vomiting
ROC	Receiver operating characteristic
SD	Standard deviation
