# Supplementary material for: Preoperative Cognitive Function and Physical Frailty Predict Decision Satisfaction and Postoperative Adherence in Older Gynecologic Oncology Patients: A Prospective Observational Study
Source: Curr Oncol. 2026 Feb 17;33(2):118. doi: 10.3390/curroncol33020118 (PMC12939682; doi:10.3390/curroncol33020118)
Supplement: Supplementary file 1 [file curroncol-33-00118-s001.zip › curroncol-4119049-supplementary.pdf]

Table S1: Scoring rubric for the postoperative adherence domain.

Domain	Score 1	Score 2	Score 3	Score 4	Score 5
1. Triflow exercise frequency	Never performed	Performed for 1–2 days in total	Performed on 3–4 days, inconsistently	Performed on most days, with 1–2 sets per day	Performed daily, ≥3 sets per day
2. Walking habits	No walking	Occasional short walks (<2 times/day)	Walks twice daily	Walks three times daily but not sustained	Walks >3 times/day, sustained and purposeful
3. Regular medication use	No intake or severe non-adherence	Misses >50% of prescribed doses	Misses 25–50% of doses	Misses <25% of doses	Fully adherent to prescribed regimen
4. Self-care independence	Fully dependent	Requires assistance for most activities of daily living	Requires assistance for some activities of daily living	Requires minimal or standby assistance	Fully independent in all activities of daily living
5. Pace of return to daily activities	No return to usual activities	Minimal return (<25% of preoperative level)	Partial return (25–50%)	Significant return (50–75%)	Full or near-full return (>75%)

ADLs: Activities of Daily Living (e.g., bathing, dressing, toileting, feeding).
